# Granule of BU-XIN RUAN-MAI Attenuates the Patients' Angina Pectoris of Coronary Heart Disease via Regulating miR-542-3p/GABARAP Signaling

**DOI:** 10.1155/2019/1808419

**Published:** 2019-12-26

**Authors:** Dong Yan, Li-li Zhao, Bo-wen Yue, Hui Qian, Zi-han Zhang, Ning Wang, Shi-hai Yan, Yu-liang Qian

**Affiliations:** ^1^Department of Pharmacology, Affiliated Hospital of Nanjing University of TCM, Nanjing, China; ^2^Nanjing University of TCM, Nanjing, China

## Abstract

**Objective:**

Coronary heart disease (CHD) has been regarded as a serious and common disease in the modern society. This study aims to investigate the effect of Granule of BU-XIN RUAN-MAI (BXRM) on angina pectoris of coronary heart disease and to explore the molecular mechanisms underlying Granule of BU-XIN RUAN-MAI-mediated protective activity against this disease.

**Methods:**

The effects of Granule of BU-XIN RUAN-MAI on clinical symptoms of patients' angina were indicated by hemorheology indicators including high shear of blood viscosity, low shear of blood viscosity, plasma viscosity, erythrocyte rigidity index, D-D dimer, fibrinogen content, and lipid content. The effects of Granule of BU-XIN RUAN-MAI on isoprenaline-induced myocardial cell injury were determined by conducting H&E staining and by performing ELISA to examine the serum content of MDA, SOD, Na^+^/K^+^-ATPase, cAMP, and the content of inflammatory factors in isoprenaline-induced rats. Meanwhile, western blot and real time PCR were used to determine the expression of genes involved in oxidation and energy metabolism, and real time PCR was also used for determination of miR-542-3p expression. Luciferase reporter assay was conducted to test the binding sites of miR-542-3p on GABARAP 3′UTR. The chemical compositions of Granule of BU-XIN RUAN-MAI were determined by liquid LC-QTOF-MS.

**Results:**

Granule of BU-XIN RUAN-MAI significantly attenuated the clinical symptoms of patients' angina by improving the patients' heart rate and by decreasing the level of hemorheology indicators and also by reducing the serum content of TC, TG, LDL, and elevated HDL content. H&E staining demonstrated that Granule of BU-XIN RUAN-MAI ameliorated the myocardial ischemia in a dose-dependent manner. Besides, Granule of BU-XIN RUAN-MAI downregulated serum MDA content and upregulated the content of SOD, Na^+^/K^+^-ATPase, and cAMP in isoprenaline-induced rats. Granule of BU-XIN RUAN-MAI significantly improved oxidation stress by increasing PPAR*α* expression, and it inhibited inflammation by downregulating expression and contents of IL-6, IL-1*β*, and TNF-*α*. Then, Granule of BU-XIN RUAN-MAI-containing serum increased the SOD content, and reduced the MDA content in angiotensin II-stimulated HUVEC cells. The granule of BU-XIN RUAN-MAI-containing serum obviously downregulated protein expressions of P40phox, P47phox, and P67phox in plasma membrane, and it significantly increased protein levels of P40phox, P47phox, and P67phox in the cytoplasm of HUVEC cells. Furthermore, GABARAP was reduced in heart tissues of ISO-induced rats and in angiotensin II-stimulated cell lines, and GABARAP was required for the inhibitory activity of Granule of BU-XIN RUAN-MAI on oxidation and inflammation *in vivo* and *in vivo*. GABARAP could be upregulated by Granule of BU-XIN RUAN-MAI by inhibiting the expression of miR-542-3p, which may significantly enhance oxidation and inflammation by targeting GABARAP in cardiomyocytes. Moreover, the silencing of GABARAP could obviously reverse the granule of BU-XIN RUAN-MAI-mediated protective activity against coronary heart disease, and interfering GABARAP expression also could partly block the anti-miR-542-3p-controlled oxidation and inflammation in cardiomyocytes. Besides, *salidroside*, *loganin*, and *polydatin* were the main compounds of granules of BU-XIN RUAN-MAI.

**Conclusion:**

Granule of BU-XIN RUAN-MAI is an excellent prescription for treatment of coronary heart disease by suppressing inflammation and NAPDH-mediated oxidative stress. The miR-542-3p/GABARAP axis is required for Granule of BU-XIN RUAN-MAI, exhibiting its protective activity against the pectoris of coronary heart disease.

## 1. Introduction

Coronary heart disease (CHD) has been regarded as a serious and common disease in the modern society [1]. A variety of pathophysiological dysfunctions has been observed in patients with coronary heart disease, including lipid content [2], oxidative stress, autonomic system dysfunction [3], inflammation [4], genetic susceptibility, smooth muscle hypercontraction [5], and endothelial dysfunction [6]. In recent years, although the western medical technology is used to reduce the mortality rate of acute coronary heart disease, it fails to improve the high incidence and poor prognosis of this disease. Angina pectoris is a major cause of disability worldwide, and angina pectoris is mainly caused by coronary artery disease or atherosclerosis [7]. Previous findings showed that there are treatment options for coronary artery disease, including medical treatment (cholesterol-lowering medications [8, 9], aspirin [10], beta blockers [11], ranolazin [12], nitroglycerin [13], calcium channel blockers [14]), coronary interventions (angioplasty and coronary stent), and coronary artery grafting [15].

Ancient traditional Chinese medicine (TCM) has been practiced for over 2,000 years [16], and modern Western medicine was introduced in the 19th century. Both the Chinese medicine and Western medicine have their own understanding and common grounds about human heart diseases and its treatment [17, 18]. In our opinion, a comprehensive analysis of the pathological situation is needed to diagnose syndrome of the coronary heart disease. Syndrome research has been a difficult and hot topic in traditional Chinese medicine because it is conductive to the management of this heart failure [19], and then the specific treatment of Chinese medicine could be prepared for patients.

In this study, the Granule of BU-XIN RUAN-MAI (BXRM) has been developed by professor Shu-Hua Tang, who is one of the fourth and fifth batch of instructors on traditional Chinese medicine. This granule of BU-XIN RUAN-MAI has gained the independent intellectual property rights of Chinese government, and it has been produced for improving the angina pectoris of coronary heart disease. He has devoted himself to traditional Chinese medicine for 50 years shaping him as an excellent and experienced master on coronary heart disease following Yellow Emperor's Classic of Internal Medicine and JIN BIAN YAO LUE. In his thoughts, the key causes of angina pectoris are deficiency of both Qi and Yin and syndrome of stagnant-heat invading collaterals. During the formation of angina pectoris, asthenic cardiac Qi and Yin deficiency of heart and kidney always induce blood stagnation and blood stasis, failure of Qi-transforming fluid leading to phlegm aggregation and dysfunction of spleen in transportation resulting in phlegm turbidity. Combination blood stasis with phlegm can block the vein flow of patients. Finally, both Yin deficiency and blood stasis are going to develop endogenous heat *in vivo*. In accordance with these above cognition of Chinese medicine, Western medicine has demonstrated that coronary heart disease is a chronic inflammatory process (termed Yin deficiency with internal heat in the Chinese medicine), lipid accumulation (termed sputum in the Chinese medicine), and migration and proliferation of smooth muscle cells (termed blood stasis in the Chinese medicine) in Western medicine [2–6].

According to the above theories of coronary heart disease (angina pectoris) Chinese medicine, the Granule of BU-XIN RUAN-MAI was prepared for treatment of angina pectoris. This Granule of BU-XIN RUAN-MAI may nourish Qi and Yin to promote blood circulation for clearing internal heat and removing obstruction in collaterals. The Granule of BU-XIN RUAN-MAI contains *Rhodiola rosea* L., *Ophiopogon japonicas* (Linn. f.) Ker-Gawl., *Cornus officinalis* Sieb. et Zucc., *Whitmania pigra* Whitman, *Ginkgo biloba* L., and *Polygonum cuspidatum* Sieb. et Zucc. Importantly, products of these herbs in Western medicine exhibit perfect activity of anti-inflammation, antioxidative stress, and anti-CHD [20–31]. Consequently, the granule of BU-XIN RUAN-MAI may be considered as an effective treatment of heart diseases.

Interestingly, overexpression of GABARAP may improve the development of coronary heart disease by promoting autophagy which can attenuate the anginal pectoris of heart disease [32–44]. In our study, we investigated the effect of Granule of BU-XIN RUAN-MAI on clinical angina pectoris of coronary heart disease. Furthermore, we determined the molecular mechanism underlying Granule of BU-XIN RUAN-MAI-regulated GABARAP expression in the treatment of coronary heart disease. Additionally, the compositions of Granule of BU-XIN RUAN-MAI were detected by time-of-flight mass spectrometry.

## 2. Materials and Methods

### 2.1. Preparation of Buxin Ruanmai Granule

First, the Buxin Ruanmai (granule of BU-XIN RUAN-MAI) granule was provided by Jiang Yin Tian Jiang Pharmaceutical Corporation (Nanjing, Jiangsu, China). The granule of BU-XIN RUAN-MAI contains *Rhodiola rosea* L. (12 g), *Ophiopogon japonicas* (Linn. f.) Ker-Gawl. (20 g), *Cornus officinalis* Sieb. et Zucc. (12 g), *Whitmania pigra* Whitman (6 g), *Ginkgo biloba* L. (20 g), and *Polygonum cuspidatum* Sieb.et Zucc. (15 g). All the plants were mixed together and grinded into powder. Then, they were shaped into Granule of BU-XIN RUAN-MAI.

### 2.2. Clinical Participants

In our study, the clinical serum samples were collected. The patients in this study were collected according to the Western medicine diagnosed by the works “Diagnostic Criteria for Coronary Atherosclerotic Heart Disease (2010, in Chinese)” and “Practice internal medicine (2017, in Chinese).” Meanwhile, we also collected the patients following “Guideline for Clinical Study of Novel Chinese Medicine (third edition, 2002, in Chinese).” The patients were excluded if they met the following criteria: (1) the patients did not meet the above principles of Western and Chinese medicine; (2) they were confirmed that they subjected to acute myocardial infarction, aortic dissection, cardiac valvular disease, cardiomyopathy, and other chest pain not caused by heart disease within half a year; (3) the patients with severe hypertension, diabetes, shock, arrhythmia, severe hepatic and renal dysfunction, hematopoietic system disorder, and psychotic disease; (4) the pregnant or lactating women; and (5) allergic constitution. Then, the patients were terminated if they met these criteria: (a) the patients were collected mistakenly; (b) the patients are not conducive to the judgment of drug efficacy; (c) the patients with incomplete personal information; (d) the patients were found with severe complications or exacerbations during treatment; (e) the patients were diagnosed with severe adverse events to treatment, or they were allergic to the treatment; and (f) the patients belonged to voluntary withdrawal from the trial. Finally, 40 patients were collected from March to December in 2015 (Department of Cardiology, Jiangsu Province Hospital of Traditional Chinese Medicine). They were divided into the placebo group and Granule of BU-XIN RUAN-MAI group randomly, with 20 patients for each group.

The patients in placebo group were treated with basic Western medicine following the Guideline for the American College of Physicians (2004). Other patients in the Granule of BU-XIN RUAN-MAI group were administrated with Granule of BU-XIN RUAN-MAI (traditional Chinese medicine) as well as the same Western medicine above. The Granule of BU-XIN RUAN-MAI was administrated at the dose of one packet/patient every 12 h (per os). This Chinese medicine would be given until the end of the experiment (8 weeks). Then, we judged the treatment effectiveness, clinical syndrome score, and electrocardiogram of Granule of BU-XIN RUAN-MAI on patients with angina based on the items in “Diagnostic Criteria for Angina and Electrocardiogram in Coronary Heart Disease (1974, in Chinese)” and “Guideline for Clinical Study of Novel Chinese Medicine (third edition, 2002, in Chinese).”

### 2.3. Acute Myocardial Ischemia in Rats

The male Sprague–Dawley rats (200 ± 20 g) were purchased from Nanjing University, China. With free access to food and water, they were kept in a clean cage with 12 h/12 h light/dark cycles and at a temperature of 23 ± 1°C. This subject was approved by Jiangsu Province Hospital of Traditional Chinese Medicine. All the experimental rats were housed under the indicated conditions for four days' acclimation. Then, myocardial ischemia of rats was caused by subcutaneous injection of isoproterenol hydrochloride (ISO, 30 mg/kg body weight), dissolved in physiological saline, for two consecutive days [45, 46]. The animals were sacrificed on the 6th day of experiment. 60 rats were put into 6 groups randomly: (1) normal control (Sham group, 5% stroke-physiological saline solution, i.g.); (2) the model group (ISO injection only, 5% stroke-physiological saline solution, i.g.); (3) the positive group (valsartan, 0.027 g/kg body weight, i.g. for three days after ISO injection); (4) the post-ISO groups: each group administered with 29.2 g/kg Granule of BU-XIN RUAN-MAI for three days after ISO injection; (5) the post-ISO groups: each group administered with 14.6 g/kg Granule of BU-XIN RUAN-MAI for three days after ISO injection; (6) the post-ISO groups: each group administered with 7.2 g/kg Granule of BU-XIN RUAN-MAI for three days after ISO injection. Finally, the blood samples were obtained from the femoral arteries of rats anesthetized with diethyl ether. Serum was saved at −80°C following centrifugation at 4°C at 3500 rpm for 20 min.

### 2.4. Cell Culture and Angiotensin II-Stimulated HUVEC and H9C2 Cell Model

The cultured HUVEC or H9C2 cells (National Infrastructure of Cell Line Resource, Beijing, China) were incubated with 10^−6^ M angiotensin II (Sigma, Shanghai, China) for 4 h to establish the cell model of NAPDH-mediated oxidative stress. Then, the cells were treated with the indicated concentration of the granule of BU-XIN RUAN-MAI-containing serum for 24 h. The cells and the corresponding medium were collected for further Western analysis, ELISA assay, and real time PCR.

### 2.5. Granule of BU-XIN RUAN-MAI-Containing Serum Preparation

The 30 rats were divided into 3 groups: (1) the sham group (normal control, 5% stroke-physiological saline solution, i.g.); (2) the positive group (valsartan, 0.027 g/kg body weight, i.g. for 6 day) and this chemical was purchased from Sigma (Shanghai, China); (3) the Granule of BU-XIN RUAN-MAI groups: each group administered with 29.2 g/kg, 14.6 g/kg and 7.2 g/kg Granule of BU-XIN RUAN-MAI i.g., for 6 days. The rats were anesthetized with diethyl ether, and the blood samples were collected from the femoral arteries. The serum was inactivated at 56°C for 30 min, and then it was filtrated with 0.22 *μ*m membrane for sterilization (Sangon, Shanghai, China). The serum was preserved at −80°C following centrifugation at 4°C at 3500 rpm for 20 min.

### 2.6. Histology

The myocardial tissues were fixed in 10% paraformaldehyde buffer. The specimens were embedded in paraffin blocks, and they were subjected to sectioning and hematoxylin-eosin (H&E) staining for histological analysis using standard protocols performed as previously described [47].

### 2.7. The Enzyme-Linked Immunosorbent Assay

The effects of Granule of BU-XIN RUAN-MAI on contents of MDA, SOD, Na^+^/K^+^-ATPase, cAMP, TNF-*α*, IL-1*β* and IL-6, and TC, TG, LDL, HDL, hs-CRP, and D-Dimer, fibrinogen in serum of patients or animals, or in cytoplasm of HUVEC cells were determined with enzyme-linked immunosorbent assay (ELISA) kits according to the manufacturer's instructions (R&D Systems, Inc., 614 Mickinley Placene, MN, USA) [48].

### 2.8. MTT Assay

The cell viability or cell proliferation was determined by 1-(4,5-dimethylthiazol-2-yl)-3,5-diphenylformazan Thiazolyl blue formazan (MTT) assay. Granule of BU-XIN RUAN-MAI has been used in China for many years. Granule of BU-XIN RUAN-MAI-containing serum was prepared by our lab in Traditional Chinese Medicine of Nanjing University, and the details were showed in Section 2.5. The prepared Granule of BU-XIN RUAN-MAI-containing serum was diluted in DMEM at 1 : 1, 1 : 10, 1 : 100, and 1 : 1000. The HUVEC cells were seeded in 96-well plates at a density of 1 × 10^4^ cells per well in 0.1 mL DMEM and were exposed to increasing concentrations of Granule of BU-XIN RUAN-MAI-containing serum at the indicated concentrations in this study for 24 h. The control cells were incubated with Granule of BU-XIN RUAN-MAI-free serum. Cell viability was determined by the MTT assay (cat. no. 57360-69-7, Sigma-Aldrich, Merck KGaA). MTT was dissolved in DMSO at 5 mg/mL, and the cells were cultured in an incubator for 4 h. The absorbance of the samples was measured using a microtiter plate reader at OD490 nm and OD655 nm.

### 2.9. Dual-Luciferase Reporter Assays

At the beginning, 6 × 10^4^ cells (HUVEC or H9C2) per well were seeded in 24-well plates. The cells were cotransfected with pmirGLO-GABA-RAP-3′UTR WT (wide type) or pmirGLO-GABARAP-3′ untranslated region (UTR)-mut (mutant), reporter plasmids and Mock (negative control), miR-542-3p mimics, and anti-miR-542-3p. After 24 h transfection, we determined luciferase activity using the Dual-Luciferase Assay Kit on GloMax 20/20 Luminometer (Promega, Madison, USA).

### 2.10. Western Blotting

To prepare total protein, the myocardial tissues of model animals or HUVEC cells were lysed for 30 min with lysis buffer (20 mM sucrose, 1 mM EDTA, 20 *μ*M Tris-Cl pH7.2, 1 mM DTT, 10 mM KCl 1.5 mM MgCl_2_, 5 *μ*g/mL pepstatin A, 10 *μ*g/mL leupeptin, and 2 *μ*g/mL aprotinin). Additionally, we isolated P40phox, P47phox, and P67phox proteins in plasma membrane or cytoplasm of HUVEC cells using the kit from Sangon (Membrane, Nuclear and Cytoplasmic Protein Extraction kit, cat. no. C510002, Shanghai, China). Briefly, the cells were washed with PBS, and they were centrifuged to collect for further use. The cells were suspended with CER A buffer, and they were incubated on ice for 15 min, and then the suspension was added with CER B buffer for following incubation on ice. They were vortexed for 5 seconds, and they were centrifuged at 15,000 g at 4°C for 15 min to prepare the cytoplasmic proteins in a precooling tube. After removing the supernatant, the precipitate was suspended with NER buffer incubated on the ice for 1 h. Finally, these samples were centrifuged at 15,000 g at 4°C for 15 min to prepare the nuclear proteins (supernatant) into a new precooling tube. All these isolated protein samples were stored at −80°C.

Then, 40 *μ*g protein for each sample was used for electrophoresis on sodium dodecyl sulfate (SDS)-polyacrylamide gels. The protein bands were transferred to PVDF membrane, and these membranes were incubated overnight at 4°C with the primary antibodies and then incubated with the HRP-carried secondary antibodies following by enhanced chemiluminescence detection. Importantly, the optical density of western blot bands was analyzed by ImageJ software. The enhanced chemiluminescence reagents were bought from Pierce Biotechnology (Rockford, IL, USA). The primary antibodies against GAPDH, PPAR*α*, GABARAP, Beclin-1, and LC3-II antibodies were got from Cell Signaling Technology (Danvers, MA, USA).

### 2.11. Real-Time PCR Analysis

Total RNA was extracted using TRIzol reagent, and cDNA was produced using the Superscript III RT kit, and real-time PCR was carried out using the SYBR Green PCR Master Mix in an ABI 7500 thermal cycler. All these agents were bought from Thermo Fisher Scientific, Inc. The primers (Genscript Co., Ltd., Nanjing, China) were as follows: GAPDH (human): forward, 5′-CAC CAT CTT CCA GGA GCG AG-3′ and reverse, 5′-GCA GGA GGC ATT GCT GAT-3′; GAPDH (*Rattus norvegicus*): forward, 5′-AGT GCC AGC CTC GTC TCA TA-3′ and reverse, 5′-GAC TGT GCC GTT GAA CTT GC-3′; TNF-*α* (human): forward, 5′-GCG ACG TGG AAC TGG CAG AAG-3′ and reverse, 5′-TCC ATG CCG TTG GCC AGG AGG-3′; TNF-*α* (*Rattus norvegicus*): forward, 5′-CAT CCG TTC TCT ACC CAG CC-3′ and reverse, 5′-AAT TCT GAG CCC GGA GTT GG-3′; IL-1*β* (human): forward, 5′-TCT CAT TGT CTC GGT GCT C-3′ and reverse, 5′-CTT TCG GGA AGA GGT TTC A-3′; IL-1*β* (*Rattus norvegicus*): forward, 5′-AGG CTG ACA GAC CCC AAA AG-3′ and reverse, 5′-CTC CAC GGG CAA GAC ATA GG-3′; IL-6 (human): forward, 5′-TGG AGT CAC AGA AGG AGT GGC TA-3′ and reverse, 5′-TGA CCA CAG TGA GGA ATG TCC AC-3′; IL-6 (*Rattus norvegicus*): forward, 5′‐TGG AGT CAC AGA AGG AGT GGC TA-3′ and reverse, 5′-TGA CCA CAG TGA GGA ATG TCC AC-3′; SOD1 (human): forward, 5′-GCA GAT GAC TTG GGC AAA GG-3′ and reverse, 5′-GGC CTC AGA CTA CAT CCA AGG-3′; SOD1 (*Rattus norvegicus*): forward, 5′-GAG GCA TGT TGG AGA CCT GG-3′ and reverse, 5′-TCG TGG ACC ACC ATA GTA CG-3′; PPAR*α* (human): forward, 5′- -3′ and reverse, 5′- -3′; PPAR*α* (human): forward, 5′-AGC TGT CAC CAC AGT AGC TT-3′ and reverse, 5′-CTT CCA GAA CTA TCC TCG CCG-3′; and PPAR*α* (*Rattus norvegicus*): forward, 5′-TCT GAA CAT TGG CGT TCG CAG-3′ and reverse, 5′-CTC GTG TGC CCT CCC TCA AG-3′. In this study, the real-time PCR was performed based on following conditions: initial denaturation of 94°C for 5 min and then 40 cycles of 94°C for 30 sec and 58°C for 30 sec and final extension of 72°C for 15 sec. Real-time PCR primers for miR-542-3p (human) are forward, 5′-TGT GAC AGA TTG ATA AC-3′ and reverse, 5′-GTG CAG GGT CCG AGG T-3′, and reverse-transcription PCR primers for miR-542-3p (human) are 5′-GTC GTA TCC AGT GCA GGG TCC GAG GTA TTC GCA CTG GAT ACG ACT TTC AG-3′. Real-time PCR primers for U6 (human) are forward, 5′-CTC GCT TCG GCA GCA CA-3′ and reverse, 5′-AAC GCT TCA CGA ATT TGC GT-3′, and reverse-transcription PCR primers for U6 (human) are 5′-GTC GTA TCC AGT GCA GGG TCC GAG GTA TTC GCA CTG GAT ACG ACA AAT ATG-3′. In our study, each sample was duplicated. We used GAPDH or U6 as an internal control for mRNA or miRNAs, and relative RNA level was calculated using the 2^−∆∆Cq^ method (17).

### 2.12. Statistical Analysis

All the data are showed as the mean ± standard deviation. We compared two groups using Student's *t*-test, and the comparisons among multiple groups were performed with one-way analysis of variance followed by Tukey's honestly significant difference test. Finally, we used SPSS 17.0 software to conduct statistical analyses (SPSS Inc., Chicago, IL, USA), and *P* < 0.05 was significant.

## 3. Result

### 3.1. The Chemical Compounds in Granule of BU-XIN RUAN-MAI and Granule of BU-XIN RUAN-MAI Alleviate the Clinical Symptoms of Angina Pectoris Diagnosed by Expert of Chinese Medicine

In this study, we observed that salidroside (275 nm), loganin (240 nm), polydatin (306 nm), resveratrol (306 nm), and emodin (280 nm) were contained in Granule of BU-XIN RUAN-MAI by using high-performance liquid chromatography (HPLC). In this study, 40 patients were collected from Jiangsu Province Hospital of TCM (traditional Chinese medicine). There were 50.00% males (10) and 50.00% (10) females in the placebo group, and 40.00% (8) males and 60.00% (12) females in the Granule of BU-XIN RUAN-MAI-treatment group (Table 1). They were randomly divided into the placebo and Granule of BU-XIN RUAN-MAI group, with 20 patients for each group. Patients' age and sex of the two groups showed no statistical difference (Table 1).

To determine the effect of Granule of BU-XIN RUAN-MAI on treatment of patients with angina pectoris, the patients in the placebo group (also named as the control group) were administrated with Western medicine, and the patients in the Granule of BU-XIN RUAN-MAI group were administrated with both Granule of BU-XIN RUAN-MAI and the same Western medicine. The total effective percentage was 90% when patients were administrated with both Western medicine and Granule of BU-XIN RUAN-MAI (Table 2). However, the total percentage was 65.00% when patients were treated with Western medicine alone (Table 2). Consequently, Granule of BU-XIN RUAN-MAI may obviously improve the angina pectoris of patients.

Then, no statistical difference existed in symptom scores of pretreatment between placebo and Granule of BU-XIN RUAN-MAI groups (Table 3). The symptom score of posttreatment patients was significantly reduced in the placebo group as well as in the Granule of BU-XIN RUAN-MAI group (^#^*P* < 0.05 versus the pretreatment patients in the placebo or Granule of BU-XIN RUAN-MAI group) (Table 3). Meanwhile, significant difference was found in symptom scores of posttreatment between the placebo and Granule of BU-XIN RUAN-MAI groups (^Δ^*P* < 0.05 versus the placebo group after treatment) (Table 3). Thus, Granule of BU-XIN RUAN-MAI may be a good choice for treatment of the patients.

### 3.2. Granule of BU-XIN RUAN-MAI Improves Patients' Heart Rate

To evaluate the effect of Granule of BU-XIN RUAN-MAI on patients' heart rate, the electrocardiogram was determined. The total effective percentage was 70.00% in the placebo group, and the total effective rate was 95% in the Granule of BU-XIN RUAN-MAI group (Table 4). The clinical curative effect of angina pectoris between the placebo and Granule of BU-XIN RUAN-MAI groups was statistically significant (Table 4). Therefore, Granule of BU-XIN RUAN-MAI may obviously better the heart rate of patients.

### 3.3. Effect of Granule of BU-XIN RUAN-MAI on Patients' Serological Indicators

No difference was found in the blood lipid level between the placebo and Granule of BU-XIN RUAN-MAI group before treatment was administrated (*P* > 0.05) (Table 5). Compared with the pretreatment patients, TC, TG, LDL, and HDL contents were significantly lower in posttreatment patients of the placebo or Granule of BU-XIN RUAN-MAI groups (Table 5). Compared with the posttreatment patients in placebo group, the TC, TG, LDL, and HDL contents were significantly reduced in the Granule of BU-XIN RUAN-MAI-administrated group (Table 5).

Furthermore, no difference was found in hemorheology indicators between the placebo and Granule of BU-XIN RUAN-MAI group before treatment was performed (*P* > 0.05) (Table 6). Compared with the pretreatment patients in the placebo, high shear of blood viscosity, low shear of blood viscosity, plasma viscosity, and erythrocyte rigidity index were downregulated when treatment was administrated (^#^*P* < 0.05) (Table 6). Compared with the posttreatment patients in the placebo group, all the indicators were significantly reduced in the Granule of BU-XIN RUAN-MAI group (Table 6).

Next, no difference was found about hs-CRP content between the placebo and Granule of BU-XIN RUAN-MAI group before treatment was administrated (*P* > 0.05) (Table 7). Compared with the pretreatment patients, the hs-CRP content was significantly downregulated after treatment in the placebo or Granule of BU-XIN RUAN-MAI group, respectively (^#^*P* < 0.05) (Table 7). Compared with the posttreatment patients in placebo group, the hs-CRP content was significantly decreased in the Granule of BU-XIN RUAN-MAI-administrated patients (^Δ^*P* < 0.05) (Table 7).

In order to determine the patients' coagulation, we found that compared with the pretreatment patients in the Granule of BU-XIN RUAN-MAI groups, the D-Dimer and fibrinogen contents were downregulated after treatment (^#^*P* < 0.05), and compared with the pretreatment patients, the D-Dimer and fibrinogen contents were also significantly decreased in the placebo groups (^#^*P* < 0.05) (Table 8). Moreover, in the Granule of BU-XIN RUAN-MAI-administrated group, the D-Dimer and fibrinogen contents were significantly downregulated compared with the placebo group (^Δ^*P* < 0.05) (Table 8).

### 3.4. Granule of BU-XIN RUAN-MAI Attenuates the Oxidation and Inflammation in Isoprenaline-Caused Myocardial Ischemia of Rats

**Figure 1:**
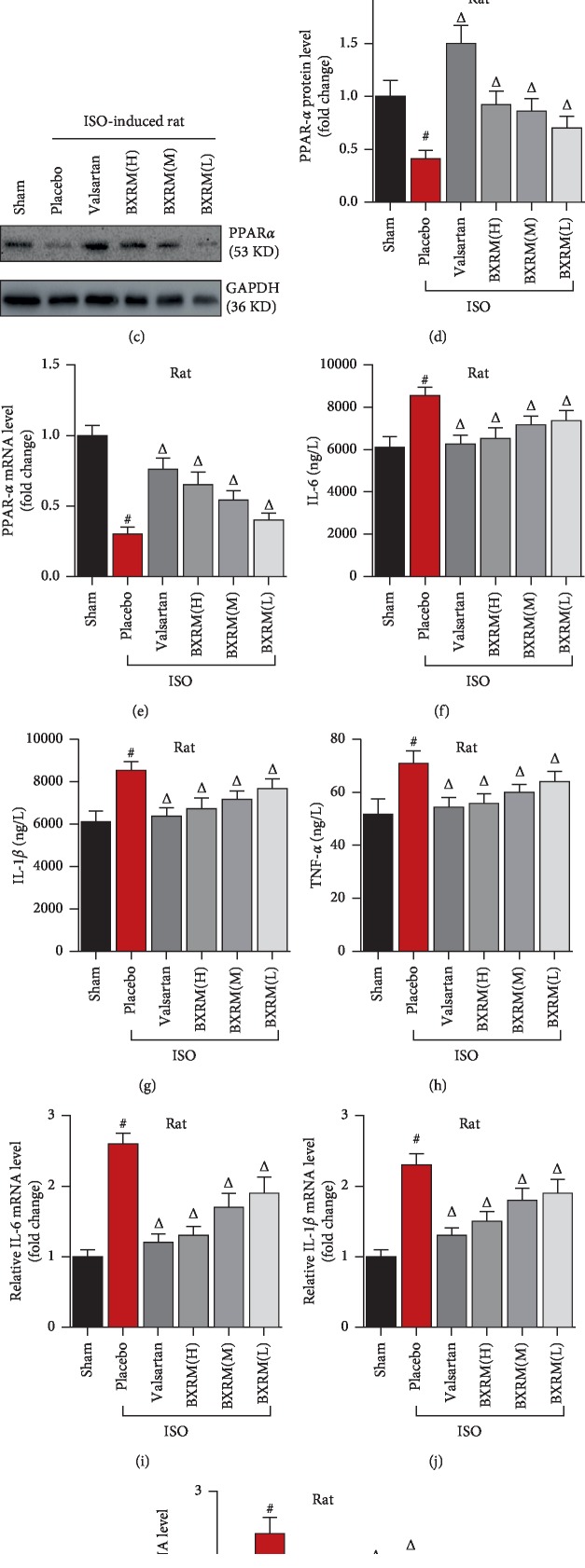
Granule of BU-XIN RUAN-MAI improves the oxidation and inflammation in myocardial ischemia of rats caused by isoprenaline. (a) Granule of BU-XIN RUAN-MAI attenuated myocardial cell injury in rats demonstrated by H&E staining. (b) The effects of Granule of BU-XIN RUAN-MAI on the content of MDA (*μ*mol/L), SOD (U/mL), Na+/K + -ATPase (*μ*mol/g protein), and cAMP (pmol/L); data shown are mean ± SD; ^#^*P* < 0.05 versus the normal group (sham) and ^Δ^*P* < 0.05 versus the model group (ISO). (c) Granule of BU-XIN RUAN-MAI significantly enhanced the PPAR*α* protein expression analyzed by Western blot, and (d) the corresponding semi-quantitative analysis was based on optical density with ImageJ software; data shown are mean ± SD; ^#^*P* < 0.001 versus the sham group and ^Δ^*P* < 0.05 versus the model group. (e) Granule of BU-XIN RUAN-MAI significantly increased the PPAR*α* mRNA expression determined by real-time PCR; data shown are mean ± SD; ^#^*P* < 0.001 versus the sham group and ^Δ^*P* < 0.05 versus the model group. (f, g, h) Granule of BU-XIN RUAN-MAI elevated the content of IL-6, IL-1*β*, and TNF-*α* detected by ELISA; data shown are mean ± SD; ^#^*P* < 0.001 versus the sham group and ^Δ^*P* < 0.05 versus the model group. (i, j, k) Granule of BU-XIN RUAN-MAI enhanced the IL-6, IL-1*β*, and TNF-*α* mRNA expressions analyzed by real-time PCR; data shown are mean ± SD; ^#^*P* < 0.001 versus the sham group and ^Δ^*P* < 0.05 versus the model group. The data are representative of three independent experiments.

To test the effect of Granule of BU-XIN RUAN-MAI on myocardial cell injury caused by isoprenaline, H&E staining was used. The results showed that the myocardial cells were seriously injured in cardiac muscles of isoprenaline-induced rats (Figure 1(a)). Importantly, valsartan may significantly inhibit the myocardial cell injury, and Granule of BU-XIN RUAN-MAI also ameliorated this dysfunction at a dose-dependent manner (Figure 1(a)). Moreover, Granule of BU-XIN RUAN-MAI as well as valsartan obviously downregulated the MDA content in serum (Figure 1(b)). We also found that Granule of BU-XIN RUAN-MAI can increase the SOD, Na^+^/K^+^-ATPase, and cAMP contents in serum of isoprenaline-induced rats (Figure 1(b)).

On the other hand, Granule of BU-XIN RUAN-MAI significantly upregulated the PPAR*α* protein expression and mRNA expression in myocardial ischemia of rats caused by isoprenaline (Figures 1(c)–1(e)). Granule of BU-XIN RUAN-MAI showed anti-inflammation activity *in vivo*. Granule of BU-XIN RUAN-MAI remarkably elevated the IL-6, IL-1*β*, and TNF-*α* contents evidenced by ELISA (Figures 1(f)–1(h)). Additionally, Granule of BU-XIN RUAN-MAI increased the IL-6, IL-1*β*, and TNF-*α* mRNA expression (Figures 1(i)–1(k)).

### 3.5. Granule of BU-XIN RUAN-MAI Improves the Angiotensin II-Stimulated Oxidative Stress in HUVEC Cells

**Figure 2:**
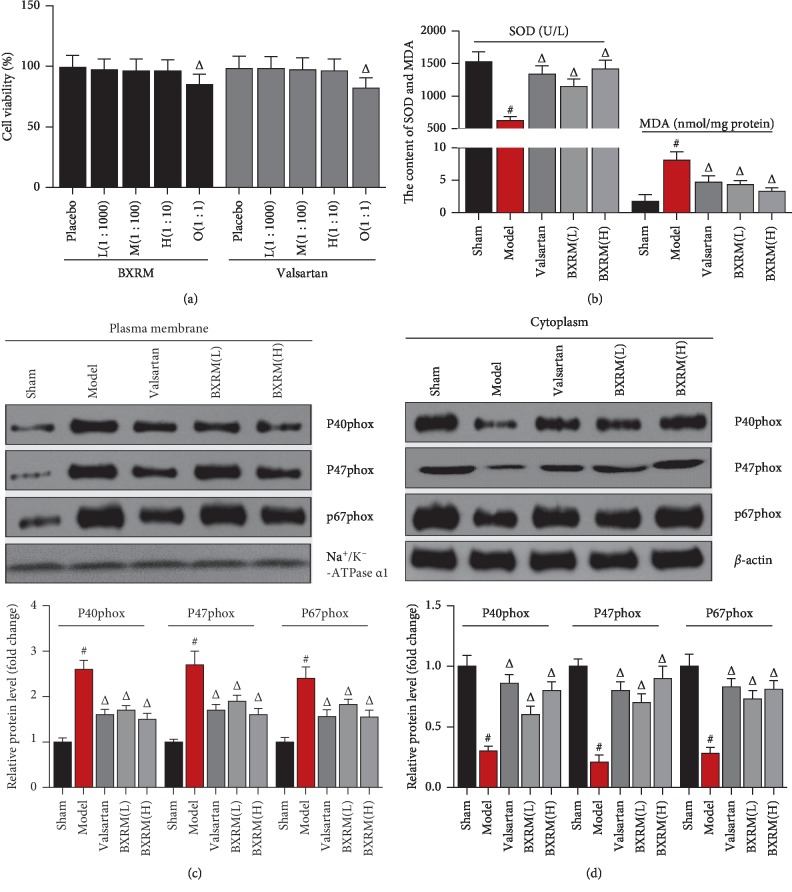
Effect of Granule of BU-XIN RUAN-MAI-containing serum on antioxidation and energy metabolism in angiotensin II-stimulated HUVEC cells. (a) The effect of Granule of BU-XIN RUAN-MAI on HUVEC cell viability. Data shown are mean ± SD; ^#^*P* < 0.001 versus the placebo group. (b) Effect of Granule of BU-XIN RUAN-MAI-containing serum on the SOD content and MDA content in angiotensin II-stimulated HUVEC cells; data shown are mean ± SD; ^#^*P* < 0.001 versus the sham group and ^Δ^*P* < 0.05 versus the model group. (c) Effect of Granule of BU-XIN RUAN-MAI-containing serum on energy metabolism indicators (P40phox, P47phox, and P67phox) at the cell membrane detected by western blot in angiotensin II-incubated HUVEC cells and the corresponding semi-quantitative analysis was based on optical density with ImageJ software; data shown are mean ± SD; ^#^*P* < 0.001 versus the sham group and ^Δ^*P* < 0.05 versus the model group. (d) Effect of Granule of BU-XIN RUAN-MAI-containing serum on energy metabolism indicators (P40phox, P47phox, and P67phox) in the cytoplasm detected by western blot in angiotensin II-incubated HUVEC cells, and the corresponding semi-quantitative analysis was based on optical density with ImageJ software; data shown are mean ± SD; ^#^*P* < 0.001 versus the sham group and ^Δ^*P* < 0.05 versus the model group. The data are representative of three independent experiments.

To determine the effect of Granule of BU-XIN RUAN-MAI-containing serum on cell oxidation and cell energy metabolism, the HUVEC cells were stimulated by angiotensin II. Firstly, the indicated concentration of Granule of BU-XIN RUAN-MAI-containing serum as well as valsartan exhibited no toxicity on HUVEC cells (Figure 2(a)). Granule of BU-XIN RUAN-MAI-containing serum and valsartan may significantly increase the SOD content and reduce the MDA content in angiotensin II-stimulated HUVEC cells (Figure 2(b)). Proteins including P40phox, P47phox, and P67phox were isolated from plasma membrane or cytoplasm of HUVEC cells using the protein extraction kit from Sangon (Membrane, Nuclear and Cytoplasmic Protein Extraction kit, cat. no. C510002, Shanghai, China). Secondly, Granule of BU-XIN RUAN-MAI-containing serum obviously downregulated P40phox, P47phox, and P67phox protein levels in the plasma membrane of HUVEC cells (Figure 2(c)). Expectedly, Granule of BU-XIN RUAN-MAI-containing serum significantly restored protein expressions of P40phox, P47phox, and P67phox in the cytoplasm of HUVEC cells (Figure 2(d)).

### 3.6. GABARAP Is Required for the Inhibitory Activity of Granule of BU-XIN RUAN-MAI on Oxidation and Inflammation In Vivo and In Vivo

**Figure 3:**
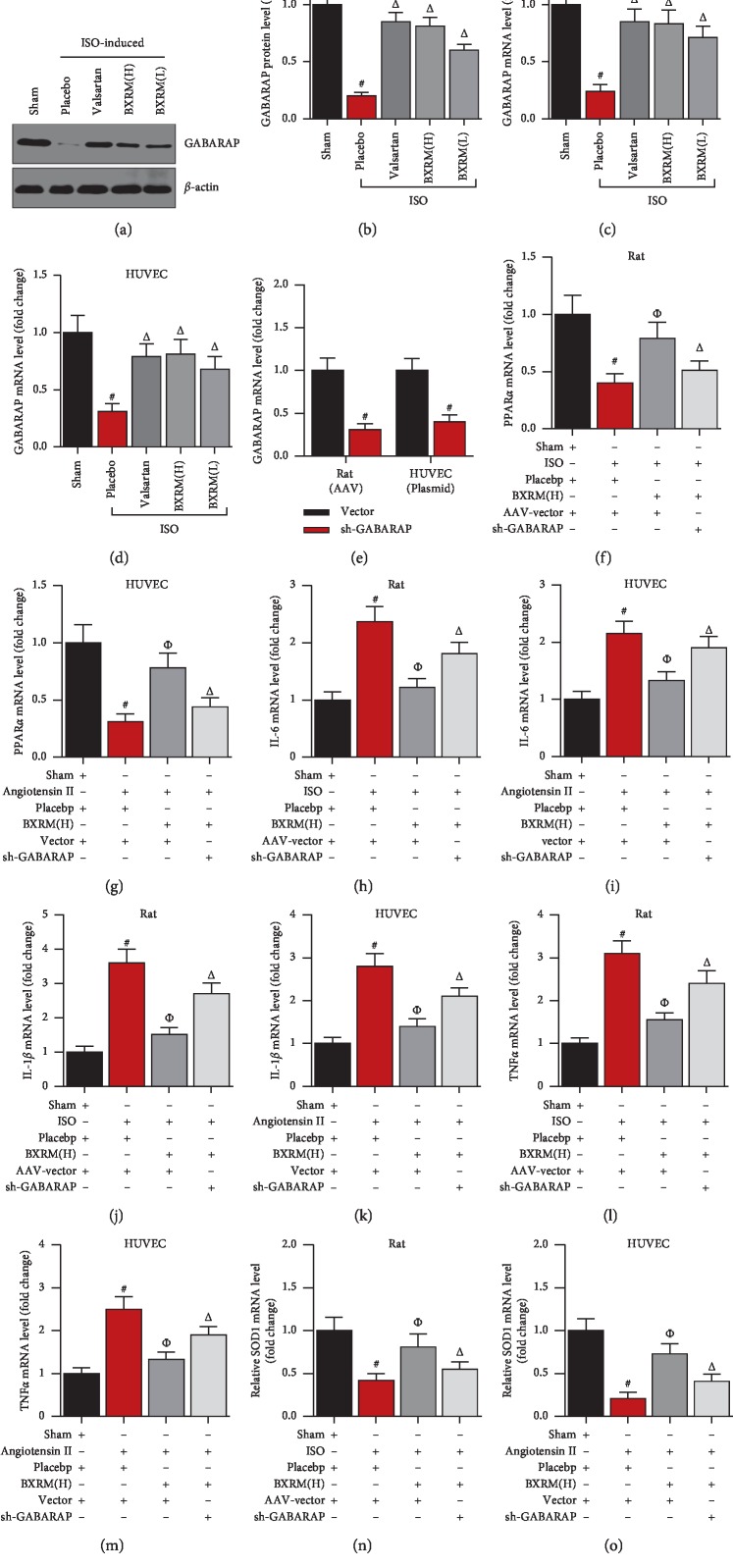
GABARAP is required for the inhibitory activity of Granule of BU-XIN RUAN-MAI on oxidation and inflammation *in vivo* and *in vivo*. (a, b) Granule of BU-XIN RUAN-MAI increased the GABARAP protein expression in heart tissues of ISO-induced rats; ^#^*P* < 0.001 versus the sham group and ^Δ^*P* < 0.05 versus the model group. (c) Granule of BU-XIN RUAN-MAI increased the GABARAP mRNA expression in heart tissues of ISO-induced rats after administration with Granule of BU-XIN RUAN-MAI for 6 days; ^#^*P* < 0.001 versus the sham group and ^Δ^*P* < 0.05 versus the model group. (d) Granule of BU-XIN RUAN-MAI increased the GABARAP mRNA expression in angiotensin II-induced HUVEC; ^#^*P* < 0.001 versus the sham group and ^Δ^*P* < 0.05 versus the placebo group. (e) Real-time PCR analysis of the GABARAP expression in rat heart and HUVEC cells after knockdown of GABARAP for 48 h; ^#^*P* < 0.001 versus the AAV-vector group or vector plasmid. (f) Real-time PCR analysis of PPAR*α* expression in rat heart after injection of adeno-associated virus followed by administration with Granule of BU-XIN RUAN-MAI for 6 days; ^#^*P* < 0.001 versus the sham + placebo + AAV-vector and ^Δ^*P* < 0.05 versus the ISO + placebo + AAV-vector. (g) Real-time PCR analysis of PPAR*α* expression in HUVEC after transfection for 48 h followed by Granule of BU-XIN RUAN-MAI incubation for 24 h; ^#^*P* < 0.001 versus the sham + placebo + vector, and ^Δ^*P* < 0.05 versus the angiotensin II + placebo + vector. (h) Real-time PCR analysis of IL-6 expression in rat heart after injection of adeno-associated virus followed by administration with Granule of BU-XIN RUAN-MAI for 6 days; ^#^*P* < 0.001 versus the sham + placebo + AAV-vector and ^Δ^*P* < 0.05 versus the ISO + placebo + AAV-vector. (i) Real-time PCR analysis of IL-6 expression in HUVEC after transfection for 48 h followed by Granule of BU-XIN RUAN-MAI incubation for 24 h; ^#^*P* < 0.001 versus the sham + placebo + vector and ^Δ^*P* < 0.05 versus the angiotensin II + placebo + vector. (j) Real-time PCR analysis of IL-1*β* expression in rat heart after injection of adeno-associated virus followed by administration with Granule of BU-XIN RUAN-MAI for 6 days; ^#^*P* < 0.001 versus the sham + placebo + AAV-vector and ^Δ^*P* < 0.05 versus the ISO + placebo + AAV-vector. (k) Real-time PCR analysis of IL-1*β* expression in HUVEC after transfection for 48 h followed by Granule of BU-XIN RUAN-MAI incubation for 24 h; ^#^*P* < 0.001 versus the sham + placebo + vector and ^Δ^*P* < 0.05 versus the angiotensin II + placebo + vector. (l) Real-time PCR analysis of TNF*α* expression in rat heart after injection of adeno-associated virus followed by administration with Granule of BU-XIN RUAN-MAI for 6 days; ^#^*P* < 0.001 versus the sham + placebo + AAV-vector and ^Δ^*P* < 0.05 versus the ISO + placebo + AAV-vector. (m) Real-time PCR analysis of TNF*α* expression in HUVEC after transfection for 48 h followed by Granule of BU-XIN RUAN-MAI incubation for 24 h; ^#^*P* < 0.001 versus the sham + placebo + vector and ^Δ^*P* < 0.05 versus the angiotensin II + placebo + vector. (n) Real-time PCR analysis of SOD1 expression in rat heart after injection of adeno-associated virus followed by administration with Granule of BU-XIN RUAN-MAI for 6 days; ^#^*P* < 0.001 versus the sham + placebo + AAV-vector and ^Δ^*P* < 0.05 versus the ISO + placebo + AAV-vector. (o) Real-time PCR analysis of SOD1 expression in HUVEC after transfection for 48 h followed by Granule of BU-XIN RUAN-MAI incubation for 24 h; ^#^*P* < 0.001 versus the sham + placebo + vector and ^Δ^*P* < 0.05 versus the angiotensin II + placebo + vector. The data are representative of three independent experiments.

To investigate the role of GABARAP in Granule of BU-XIN RUAN-MAI-mediated suppression of oxidation and inflammation, we knocked down the GABARAP expression in rat and in HUVEC cells, and then the cells were incubated with Granule of BU-XIN RUAN-MAI. Firstly, GABARAP was decreased in the heart of ISO-induced rats, whereas it was upregulated by Granule of BU-XIN RUAN-MAI (Figures 3(a)–3(c)). Similarly, GABARAP was reduced in angiotensin II-induced HUVEC cells; however, it was upregulated by Granule of BU-XIN RUAN-MAI (Figure 3(d)). Moreover, knockdown of GABARAP could significantly block the Granule of BU-XIN RUAN-MAI-controlled upregulation of PPAR*α* in ISO-induced rats and in angiotensin II-incubated HUVEC cells (Figures 3(e)–3(g)). Meanwhile, suppression of GABARAP could obviously block the Granule of BU-XIN RUAN-MAI-regulated gene expression of inflammatory factors including IL-6, IL-1*β*, and TNF *α* in rat and HUVEC cells (Figures 3(h)–3(m)). Finally, downregulation of GABARAP could significantly block the Granule of BU-XIN RUAN-MAI-controlled expression of oxidative stress-associated gene SOD1 *in vivo* and *in vitro* (Figures 3(n) and 3(o)).

### 3.7. miR-542-3p Is Involved in Granule of BU-XIN RUAN-MAI-Induced Upregulation of GABARAP Expression

**Figure 4:**
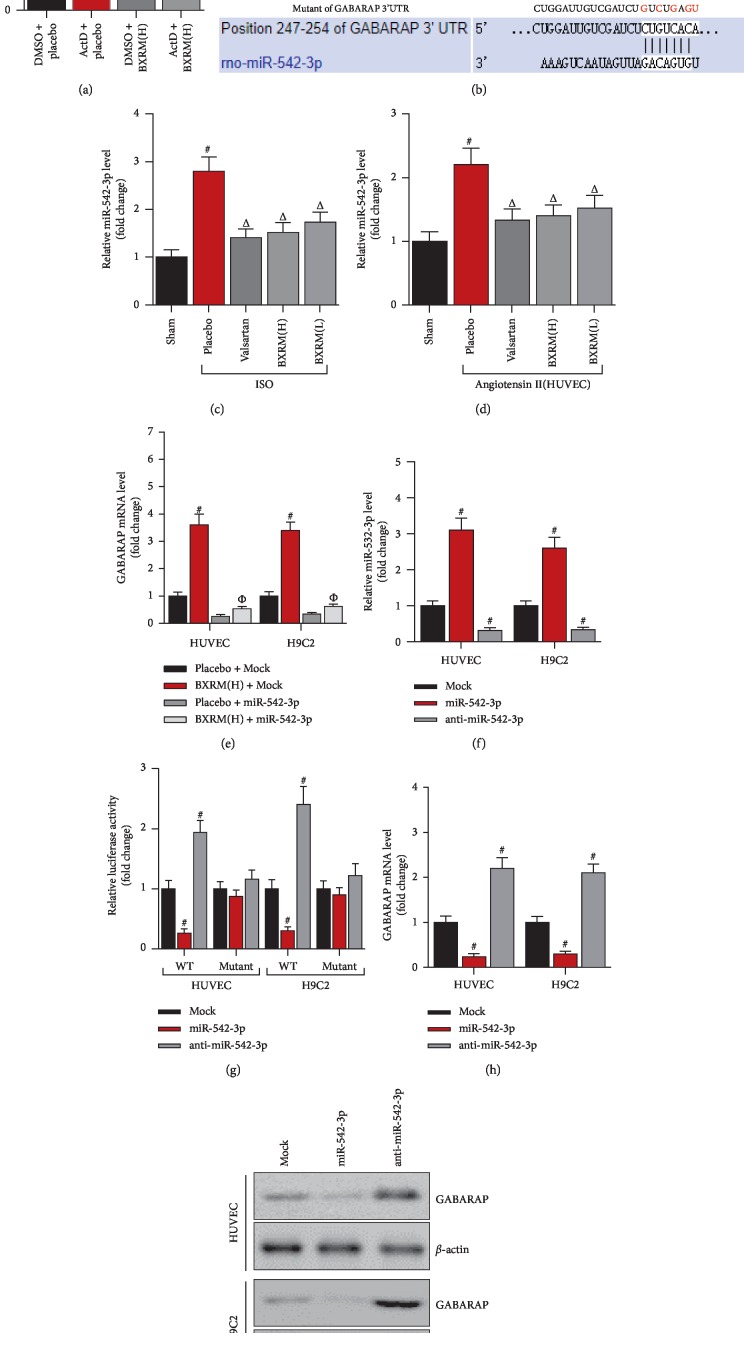
miR-542-3p is involved in Granule of BU-XIN RUAN-MAI-induced upregulation of GABARAP expression. (a) Real-time PCR of GABARAP expression in HUVEC cells and the cells were incubated with both ActD and Granule of BU-XIN RUAN-MAI for 10 h; ^#^*P* < 0.001 versus the DMSO + placebo and ^Δ^*P* < 0.05 versus DMSO + Granule of BU-XIN RUAN-MAI. (b) Predication of binding site of miR-542-3p on GABARAP 3′UTR by Targetscan (http://www.targetscan.org/vert_72/). (c) Real-time PCR analysis of miR-542-3p in rat heart after Granule of BU-XIN RUAN-MAI administration as described above; ^#^*P* < 0.001 versus the sham and ^Δ^*P* < 0.05 versus ISO + placebo. (d) Real-time PCR analysis of miR-542-3p in HUVEC after Granule of BU-XIN RUAN-MAI incubation; ^#^*P* < 0.001 versus the sham and ^Δ^*P* < 0.05 versus angiotensin II + placebo. (e) Real-time PCR analysis of GABARAP in HUVEC and H9C2 cells after transfection for 48 h followed by Granule of BU-XIN RUAN-MAI incubation for 24 h; ^#^*P* < 0.001 versus placebo + Mock and ^Δ^*P* < 0.05 versus placebo + miR-542-3p. (f) Real-time PCR analysis of GABARAP in HUVEC and H9C2 cells after transfection for 48 h; ^#^*P* < 0.001 versus Mock. (g) Luciferase reporter assay analysis of pmir-GABARAP-3′UTR luciferase activity after transfection for 48 h; ^#^*P* < 0.001 versus Mock. (h) Real-time PCR analysis of GABARAP expression after transfection for 48 h, ^#^*P* < 0.001 versus Mock. (i) Western blot analysis of GABARAP expression after transfection for 48 h. The data are representative of three independent experiments.

The above data showed that Granule of BU-XIN RUAN-MAI could significantly attenuate oxidation and inflammation in coronary heart disease by upregulating GABARAP expression. However, the regulatory molecular mechanism underlying the Granule of BU-XIN RUAN-MAI-controlled GABARAP expression remains unknown. In this study, we found that Granule of BU-XIN RUAN-MAI significantly upregulated GABARAP expression by stabilizing its 3′UTR (Figure 4(a)). In this study, GABARAP might be targeted by miR-542-3p (Figure 4(b)). Further data revealed that miR-542-3p was increased in the heart of ISO rats and in angiotensin-incubated HUVEC cells, while its level was suppressed in Granule of BU-XIN RUAN-MAI (Figures 4(c) and 4(d)). More importantly, GABARAP was upregulated by Granule of BU-XIN RUAN-MAI partly depending on miR-542-3p (Figure 4(e)). Next, miR-542-3p significantly inhibited luciferase activity of GABARAP 3′UTR, whereas suppression of miR-542-3p could enhance the luciferase activity of GABARAP 3′UTR (Figures 4(f) and 4(g)). Consistently, overexpression of miR-542-3p markedly suppressed GABARAP expression both at the mRNA and protein levels (Figures 4(h) and 4(i)).

### 3.8. miR-542-3p Promotes Oxidation and Inflammation by Targeting GABARAP in Cell Lines

**Figure 5:**
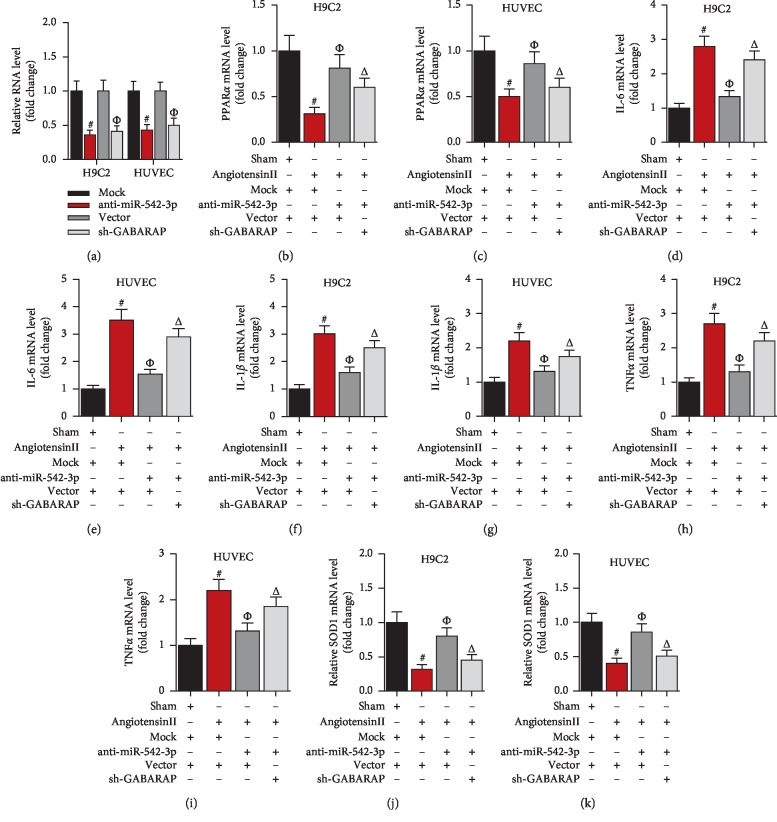
miR-542-3p regulates oxidation and inflammation by targeting GABARAP in cell lines. (a) Real-time PCR analysis of miR-542-3p or GABARAP in cell lines after transfection for 48 h; ^#^*P* < 0.001 versus Mock and ^Δ^*P* < 0.05 versus vector. (b, c) Real-time PCR analysis of PPAR*α* expression in H9C2 and HUVEC cells after 48 h transfection followed by 4 h angiotensin II-stimulation; ^#^*P* < 0.001 versus the sham + Mock + vector, ^Φ^*P* < 0.05 versus the angiotensin II + Mock + vector, and ^Δ^*P* < 0.05 versus the angiotensin II + anti-miR-542-3p + vector. (d, e) Real-time PCR analysis of IL-6 expression in H9C2 and HUVEC cells after 48 h transfection followed by 4 h angiotensin II-stimulation; ^#^*P* < 0.001 versus the sham + Mock + vector, ^Φ^*P* < 0.05 versus the angiotensin II + Mock + vector, and ^Δ^*P* < 0.05 versus the angiotensin II + anti-miR-542-3p + vector. (f, g) Real-time PCR analysis of IL-1*β* expression in H9C2 and HUVEC cells after 48 h transfection followed by 4 h angiotensin II-stimulation; ^#^*P* < 0.001 versus the sham + Mock + vector, ^Φ^*P* < 0.05 versus the angiotensin II + Mock + vector, and ^Δ^*P* < 0.05 versus the angiotensin II + anti-miR-542-3p + vector. (h, i) Real-time PCR analysis of TNF*α* expression in H9C2 and HUVEC cells after 48 h transfection followed by 4 h angiotensin II-stimulation; ^#^*P* < 0.001 versus the sham + Mock + vector, ^Φ^*P* < 0.05 versus the angiotensin II + Mock + vector, and ^Δ^*P* < 0.05 versus the angiotensin II + anti-miR-542-3p + vector. (j, k) Real-time PCR analysis of SOD1 expression in H9C2 and HUVEC cells after 48 h transfection followed by 4 h angiotensin II-stimulation; ^#^*P* < 0.001 versus the sham + Mock + vector, ^Φ^*P* < 0.05 versus the angiotensin II + Mock + vector, and ^Δ^*P* < 0.05 versus the angiotensin II + anti-miR-542-3p + vector.

To explore the role of miR-542-3p/GABARAP axis in coronary heart disease, we examined the effects of miR-542-3p/GABARAP axis on oxidation and inflammation (Figure 5). Firstly, knockdown of miR-542-3p could significantly reverse angiotensin II-induced downregulation of oxidation indicator PPAR*α*, whereas silencing of GABARAP obviously blocked anti-miR-542-3p-controlled PPAR*α* expression in H9C2 and HUVEC cells (Figures 5(a)–5(c)). Then, interfering miR-542-3p suppressed angiotensin II-mediated upregulation of inflammatory factors including IL-6, IL-1*β*, and TNF*α*, while knockdown of GABARAP markedly reversed these miR-542-3p-induced gene expressions (Figures 5(d)–5(i)). Similarly, silencing of GABARAP could significantly block the miR-542-3p-controlled expression of oxidative stress-associated genes including SOD1 in H9C2 and HUVEC cells (Figures 5(j) and 5(k)). Besides, according to our previous studies (written in Chinese) [49, 50], we analyzed the compositions of Granule of BU-XIN RUAN-MAI. LC-QTOF-MS analysis showed that *salidroside*, *loganin*, and *polydatin* were the main compounds of Granule of BU-XIN RUAN-MAI (Supporting [Supplementary-material supplementary-material-1]).

## 4. Discussion

Coronary heart disease (CHD) is a common disease nowadays [1]. The western medical technology fails to improve poor prognosis of this disease. In this study, we investigated the effect of Granule of BU-XIN RUAN-MAI, a clinical Chinese medicine, on CHD patients with angina pectoris. The data showed that Granule of BU-XIN RUAN-MAI can ameliorate the clinical coronary heart disease and inhibit isoprenaline-induced myocardial cell injury. In the end, Granule of BU-XIN RUAN-MAI may significantly trigger autophagy in myocardial cells.

Ancient traditional Chinese medicine (TCM) has been practiced for a long history [16]. Granule of BU-XIN RUAN-MAI has been prepared by professor Shu-Hua Tang, an excellent expert of traditional Chinese medicine. The Granule of BU-XIN RUAN-MAI contains *Rhodiola rosea* L., *Ophiopogon japonicas* (Linn. f.) Ker-Gawl., *Cornus officinalis* Sieb. et Zucc., *Whitmania pigra* Whitman, *Ginkgo biloba* L., *Polygonum cuspidatum* Sieb.et Zucc., and so on [20–31]. Professor Shu-Hua Tang considered that asthenic cardiac Qi and Yin deficiency of heart and kidney often occurred in the shape of coronary heart disease. Western medicine has stated that coronary heart disease is a chronic inflammatory process (Yin deficiency with internal heat), lipid accumulation (sputum) and migration and proliferation of smooth muscle cells (blood stasis). Luckily, Granule of BU-XIN RUAN-MAI is just the compound prescription that it may nourish Qi and Yin to enhance blood circulation for clearing internal heat and removing obstruction in collaterals, and products of these herbs exhibit perfect activity of anti-inflammation, antioxidative stress, antithrombotic activity, and cardiovascular protective activity [20–31, 51–56]. Consequently, Granule of BU-XIN RUAN-MAI is a perfect prescription for treatment of ischemic heart disease (angina pectoris).

In the present study, to determine the effect of Granule of BU-XIN RUAN-MAI on treatment of patients with angina pectoris, the patients in the placebo group were administrated with Western medicine, and the patients in the Granule of BU-XIN RUAN-MAI group were administrated with Western medicine and Granule of BU-XIN RUAN-MAI (traditional Chinese medicine). The data showed that patients with angina pectoris were improved more effectively by administration of Granule of BU-XIN RUAN-MAI. Furthermore, the results demonstrated that Granule of BU-XIN RUAN-MAI can keep patients' heart rate at the proper level. To investigate the effect of Granule of BU-XIN RUAN-MAI on patients' serological indicators, the data proved that Granule of BU-XIN RUAN-MAI could significantly decrease the TC, TG, LDL, and HDL contents, and Granule of BU-XIN RUAN-MAI may downregulate the patients' high shear of blood viscosity, low shear of blood viscosity, plasma viscosity, erythrocyte rigidity index, hs-CRP content, and D-Dimer and fibrinogen contents. These results suggested that Granule of BU-XIN RUAN-MAI was able to improve patients' heart rate, promote blood circulation, reduce lipid content in serum, and inhibit the formation of thrombus. Importantly, Granule of BU-XIN RUAN-MAI contained *salidroside*, *loganin*, and *polydatin* determined by LC-QTOF-MS analysis. *Salidroside* [57], *loganin* [58], and *polydatin* [59] can protect against oxidative stress, which is the main cause of coronary heart disease. Thus, Granule of BU-XIN RUAN-MAI may be considered as a good prescription for treatment of ischemic heart disease.

Similarly, we determined the effect of Granule of BU-XIN RUAN-MAI in the myocardial injury model. In isoprenaline-induced rats, Granule of BU-XIN RUAN-MAI sustained the shape of myocardial cells evidenced by H&E staining, and Granule of BU-XIN RUAN-MAI significantly inhibited the myocardial cell injury by reducing the MDA content in serum and elevating the SOD1, Na^+^/K^+^-ATPase, and cAMP content in serum. Additionally, in isoprenaline-induced rats, Granule of BU-XIN RUAN-MAI ameliorated myocardial ischemia by downregulating the inflammatory factor levels of IL-6, IL-1*β*, and TNF-*α*. These data implied that Granule of BU-XIN RUAN-MAI could improve heart functions during the myocardial ischemia.

The production of oxidative stress may be enhanced by activating the angiotensin II-associated NADPH oxidase (Nox) in endothelial cells [60]. Oxidative stress is an important factor to endothelial disfunction [61]. The NADPH oxidase family members are multicomponent protein complexes composed of catalytic subunits including Nox1-5, organizer subunits such as p47phox or Noxo1, activator subunits such as p67phox or Noxa1, and other regulatory subunits such as p22phox and p40phox and the binding partner Rac [62]. In our study, Granule of BU-XIN RUAN-MAI-containing serum obviously downregulated protein levels of P40phox, P47phox, and P67phox in the plasma membrane of HUVEC cells, and it significantly increased protein expressions of P40phox, P47phox, and P67phox in the cytoplasm of HUVEC cells. These findings suggest that Granule of BU-XIN RUAN-MAI-containing serum can downregulate the NADPH-mediated oxidative stress level in angiotensin II-stimulated HUVEC cells. Subsequently, Granule of BU-XIN RUAN-MAI can inhibit coronary heart disease by regulating NADPH-mediated oxidative stress.

GABARAP has been considered as a novel and essential regulator that it could improve coronary heart disease including angina pectoris and arteriosclerosis by increasing autophagy, which is a beneficial biological process for treatment of coronary heart disease [38–44]. Thus, we investigated whether GABARAP was involved in the Granule of BU-XIN RUAN-MAI-induced inhibition of angina pectoris. Firstly, GABARAP was decreased in the heart of ISO-induced rats and in angiotensin II-incubated HUVEC cells, whereas it was upregulated by Granule of BU-XIN RUAN-MAI, implying that GABARAP-mediated autophagy might be involved in Granule of BU-XIN RUAN-MAI alleviating heart disease. Furthermore, knockdown of GABARAP could significantly reverse the Granule of BU-XIN RUAN-MAI-controlled expression of genes including PPAR*α*, IL-6, IL-1*β*, and TNF*α*, SOD1, and MDA *in vivo* and *in vitro*, suggesting that Granule of BU-XIN RUAN-MAI shows its inhibitory activity against oxidation and inflammation in angina pectoris of heart disease partly depending on the GABARAP expression. Thus, GABARAP is required for Granule of BU-XIN RUAN-MAI exhibiting its protective effect against heart disease, and it is meaningful for us to investigate the role of GABARAP in Granule of BU-XIN RUAN-MAI against heart disease. Based on these studies, we investigated the molecular mechanisms of Granule of BU-XIN RUAN-MAI in modulating the GABARAP expression. The data demonstrated that Granule of BU-XIN RUAN-MAI upregulated GABARAP by keeping stability of GABARAP mRNA 3′UTR, suggesting that Granule of BU-XIN RUAN-MAI could increase the GABARAP expression through modulating miRNAs level.

Previous studies have pointed out that miRNAs play an important role in coronary heart disease. For example, downregulated miRNA-26a-5p enhances the apoptosis of endothelial cells in coronary heart disease by suppressing PI3K/AKT signaling [63]. MiR-590 increases endothelial cell apoptosis by inactivating TLR4/NF-*κ*B signaling in atherosclerosis [64]. Inhibition of microRNA-429 alleviates myocardial injury of rats with coronary heart disease [65]. MicroRNA-7b ameliorates ischemia/reperfusion-induced H9C2 cardiomyocyte apoptosis through the hypoxia-inducible factor-1/p-p38 pathway [66]. miR-542-3p often serves as a tumor suppressor in various cancers including epithelial ovarian cancer [67], hepatocellular carcinoma [68], osteosarcoma [69], and meanwhile, miR-542-3p promotes hepatic stellate cell activation and fibrosis by regulating BMP‐7 [70]. In our study, we observed that miR-542-3p was increased in ISO-induced rats and in angiotensin II-induced HUVEC cells, and it could be inhibited by Granule of BU-XIN RUAN-MAI. Then, miR-542-3p can significantly promote oxidation and inflammation in cardiomyocytes of coronary heart disease by targeting GABARAP. Additionally, overexpression of miR-542-3p markedly inhibited GABARAP expression while interfering miR-542-3p increased the GABARAP level in cardiomyocytes. Finally, Granule of BU-XIN RUAN-MAI upregulated the GABARAP expression partly by depending on miR-542-3p. Together, these findings indicate that miR-542-3p may aggravate oxidation and inflammation in coronary heart disease by targeting GABARAP, and miR-542-3p/GABARAP axis is required for Granule of BU-XIN RUAN-MAI showing its protective activity against angina pectoris of coronary heart disease. Further data demonstrated that knockdown of miR-542-3p-mediated inhibition of oxidation and inflammation could be partly blocked by silencing of GABARAP in cardiomyocytes.

## 5. Conclusions

The data from clinic and animal model demonstrated that Granule of BU-XIN RUAN-MAI is an excellent prescription for treatment of coronary heart disease by suppressing the inflammation and NAPDH-mediated oxidative stress. MiR-542-3p/GABARAP axis is required for Granule of BU-XIN RUAN-MAI exhibiting its protective activity against pectoris of coronary heart disease.

## Figures and Tables

**Table 1:** Patients' sex and age.

Group	Number of cases	Male	Female	Average age
Placebo	20	10	10	66.75 ± 8.10
Granule of BU-XIN RUAN-MAI	20	8	12	64.30 ± 9.69

The statistical difference between the placebo and Granule of BU-XIN RUAN-MAI group (BXRM) was tested by Student's *t* test (*P* > 0.05 versus the placebo group).

**Table 2:** Clinical curative effect of Granule of BU-XIN RUAN-MAI on angina pectoris diagnosed by experts of Chinese medicine.

Group	Number of cases	Excellence	Improvement	Failure	Effective percentage (%)
Placebo	20	2	11	7	65.00
Granule of BU-XIN RUAN-MAI	20	5	13	2	90.00^Δ^

The clinical curative effect of angina pectoris in the two groups was statistically significant (^Δ^*P* < 0.05 versus the placebo group).

**Table 3:** Effect of Granule of BU-XIN RUAN-MAI on clinical syndrome score evaluated by experts of Chinese medicine.

Group	Number of cases	Symptom score of pretreatment	Symptom score of posttreatment
Placebo	20	23.70 ± 5.13	17.75 ± 3.06^#^
Granule of BU-XIN RUAN-MAI	20	23.35 ± 4.54	10.15 ± 1.76^#Δ^

In this table, there was no statistical difference in symptom scores of pretreatment between two groups (Student's *t* test, *P* > 0.05). The ^#^*P* < 0.05 versus the pretreatment patients in the placebo or Granule of BU-XIN RUAN-MAI group; ^Δ^*P* < 0.05 versus posttreatment patients in the placebo group.

**Table 4:** Effect of Granule of BU-XIN RUAN-MAI on patients' heart rate evidenced by electrocardiogram.

Group	Number of cases	Excellence	Improvement	Failure	Heart rate (%)
Placebo	20	4	10	6	70.00
Granule of BU-XIN RUAN-MAI	20	6	13	1	95.00^Δ^

The clinical curative effect of angina pectoris in the two groups was statistically significant (Student's *t* test); ^Δ^*P* < 0.05 versus the placebo group.

**Table 5:** Effect of Granule of BU-XIN RUAN-MAI on patients' blood lipid content.

Group	Number of cases	Stage	TC	TG	LDL	HDL
Placebo	20	Pretreatment	5.91 ± 0.73	2.11 ± 0.13	3.94 ± 0.68	0.87 ± 0.12
	20	Posttreatment	4.67 ± 0.75^#^	1.64 ± 0.15^#^	3.24 ± 0.65^#^	0.56 ± 0.13^#^
Granule of BU-XIN RUAN-MAI	20	Pretreatment	5.95 ± 0.76	2.12 ± 0.14	3.92 ± 0.67	0.73 ± 0.11
	20	Posttreatment	4.10 ± 0.45^#Δ^	1.09 ± 0.13^#Δ^	2.58 ± 0.44^#Δ^	0.39 ± 0.08^#Δ^

There was no significant difference in blood lipid level between the two groups before treatment was administrated (Student's *t* test, *P* > 0.05). ^#^*P* < 0.05 versus the pretreatment patients in the placebo or Granule of BU-XIN RUAN-MAI group; ^Δ^*P* < 0.05 versus the posttreatment patients in the placebo group.

**Table 6:** Effect of Granule of BU-XIN RUAN-MAI on hemorheology indicators in patents.

Group	Number of cases	Stage	High shear of blood viscosity	Low shear of blood viscosity	Plasma viscosity	Erythrocyte rigidity index
Placebo	20	Pre-treatment	4.52 ± 0.24	7.99 ± 0.95	1.57 ± 0.17	7.84 ± 0.51
	20	Post-treatment	3.87 ± 0.27^#^	7.14 ± 0.89^#^	1.52 ± 0.16^#^	7.02 ± 0.59^#^
Granule of BU-XIN RUAN-MAI	20	Pre-treatment	4.56 ± 0.26	8.24 ± 0.98	1.61 ± 0.16	7.73 ± 0.58
	20	Post-treatment	3.25 ± 0.17^#Δ^	6.53 ± 0.87^#Δ^	1.22 ± 0.09^#Δ^	5.98 ± 0.43^#Δ^

There was no significant difference in hemorheology indicators between the placebo and Granule of BU-XIN RUAN-MAI groups before treatment was administrated (Student's *t* test, *P* > 0.05). ^#^*P* < 0.05 versus the pretreatment patients in the placebo or Granule of BU-XIN RUAN-MAI group; ^Δ^*P* < 0.05 versus the posttreatment patients in the placebo group.

**Table 7:** Effect of Granule of BU-XIN RUAN-MAI on hs-CRP level in patents.

Group	Number of cases	Stage	hs-CRP content
Placebo	20	Pretreatment	5.49 ± 0.86
	20	Posttreatment	3.76 ± 0.34^#^
Granule of BU-XIN RUAN-MAI	20	Pretreatment	5.35 ± 1.08
	20	Posttreatment	2.82 ± 0.39^#Δ^

There was no significant difference in hs-CRP content between the two groups before treatment was administrated (Student's *t* test, *P* > 0.05). ^#^*P* < 0.05 versus the pretreatment patients in the placebo or Granule of BU-XIN RUAN-MAI group; ^Δ^*P* < 0.05 versus the posttreatment patients in the placebo group.

**Table 8:** Effect of Granule of BU-XIN RUAN-MAI on indicators of patients' coagulation.

Group	Number of cases	Stage	D-Dimer	Fibrinogen
Placebo	20	Pretreatment	2.03 ± 1.12	3.97 ± 1.13
	20	Posttreatment	1.23 ± 0.86^#^	2.57 ± 1.27^#^
Granule of BU-XIN RUAN-MAI	20	Pretreatment	2.09 ± 1.08	4.01 ± 1.22
	20	Posttreatment	0.66 ± 0.42^#Δ^	1.83 ± 0.68^#Δ^

There was no significant difference in patients' coagulation indicator (D-Dimer and fibrinogen) contents between the placebo and Granule of BU-XIN RUAN-MAI group before treatment was administrated (*P* > 0.05). ^#^*P* < 0.05 versus the pretreatment patients in the placebo or Granule of BU-XIN RUAN-MAI group; ^Δ^*P* < 0.05 versus the posttreatment patients in the placebo group.

## Data Availability

The data used to support the findings of this study are available from the corresponding author upon request.

## Abbreviations

BXRM: Granule of BU-XIN RUAN-MAI
CHD: Coronary heart disease
TCM: Traditional Chinese medicine
MDA: Malondialdehyde
SOD: Superoxide dismutase
cAMP: Cyclic adenosine monophosphate
TNF-*α*: Tumor necrosis factor alpha
IL-1*β*: Interleukin-1 beta
IL-6: Interleukin-6
TC: Total cholesterol
TG: Triglyceride
LDL: Low-density lipoprotein cholesterol
HDL: High-density lipoprotein cholesterol
hs-CRP: High-sensitivity C-reactive protein
H&E: Hematoxylin and eosin staining
SDS: Sodium dodecyl sulfate
ECL: Enhanced chemiluminescence
ISO: Isoproterenol hydrochloride
ELISA: Enzyme-linked immunosorbent assay
PVDF: Polyvinylidene fluoride
PCR: Polymerase chain reaction.

## Supplementary Materials

Supplementary Materials Supporting Figure 1: the compositions of Granule of BU-XIN RUAN-MAI. In this study, the compositions of Granule of BU-XIN RUAN-MAI were determined by LC-QTOF-MS analysis. The results showed that salidroside (a), loganin (b), and polydatin (c) were the main compounds of Granule of BU-XIN RUAN-MAI granule.
