# Supplementary material for: Granule of BU-XIN RUAN-MAI Attenuates the Patients' Angina Pectoris of Coronary Heart Disease via Regulating miR-542-3p/GABARAP Signaling
Source: Evid Based Complement Alternat Med. 2019 Dec 26;2019:1808419. doi: 10.1155/2019/1808419 (PMC6948311; doi:10.1155/2019/1808419)

**Supporting methods and materials.**

The Buxin Ruanmai (Granule of BU-XIN RUAN-MAI) granule was provided by Jiang Yin Tian Jiang Pharmaceutical Coporation (Nanjing, Jiangsu, China). The Granule of BU-XIN RUAN-MAI contains *Rhodiola rosea* L. (12 g), *Ophiopogon japonicas* (Linn. f.) Ker-Gawl. (20 g), *Cornus officinalis* Sieb. et Zucc. (12 g), *Whitmania pigra* Whitman (6 g), *Ginkgo biloba* L*.* (20 g)*, Polygonum cuspidatum* Sieb.et Zucc. (15 g). All the herbs were mixed together and grinded into powder, and they were shaped into Granule of BU-XIN RUAN-MAI. To determine the chemical compositions of Granule of BU-XIN RUAN-MAI, we performed liquid chromatography/quadrupole time-of-flight mass spectrometry (**LC-QTOF-MS**) *at Nanjing University of Traditional Chinese* Medicine. The reference standards, *salidroside,* *Loganin, polydatin*, ethanol and all the other reagents were purchased from Sigma-Aldrich *(St. Louis, MO, USA).*

**Supporting Figure 1. The compositions of Granule of BU-XIN RUAN-MAI.** In this study, the compositions of Granule of BU-XIN RUAN-MAI granule were determined by LC-QTOF-MS analysis. The results showed that *salidroside* (**a**)*,* *Loganin* (**b**)*, polydatin* (**c**) were the main compounds of Granule of BU-XIN RUAN-MAI granule.


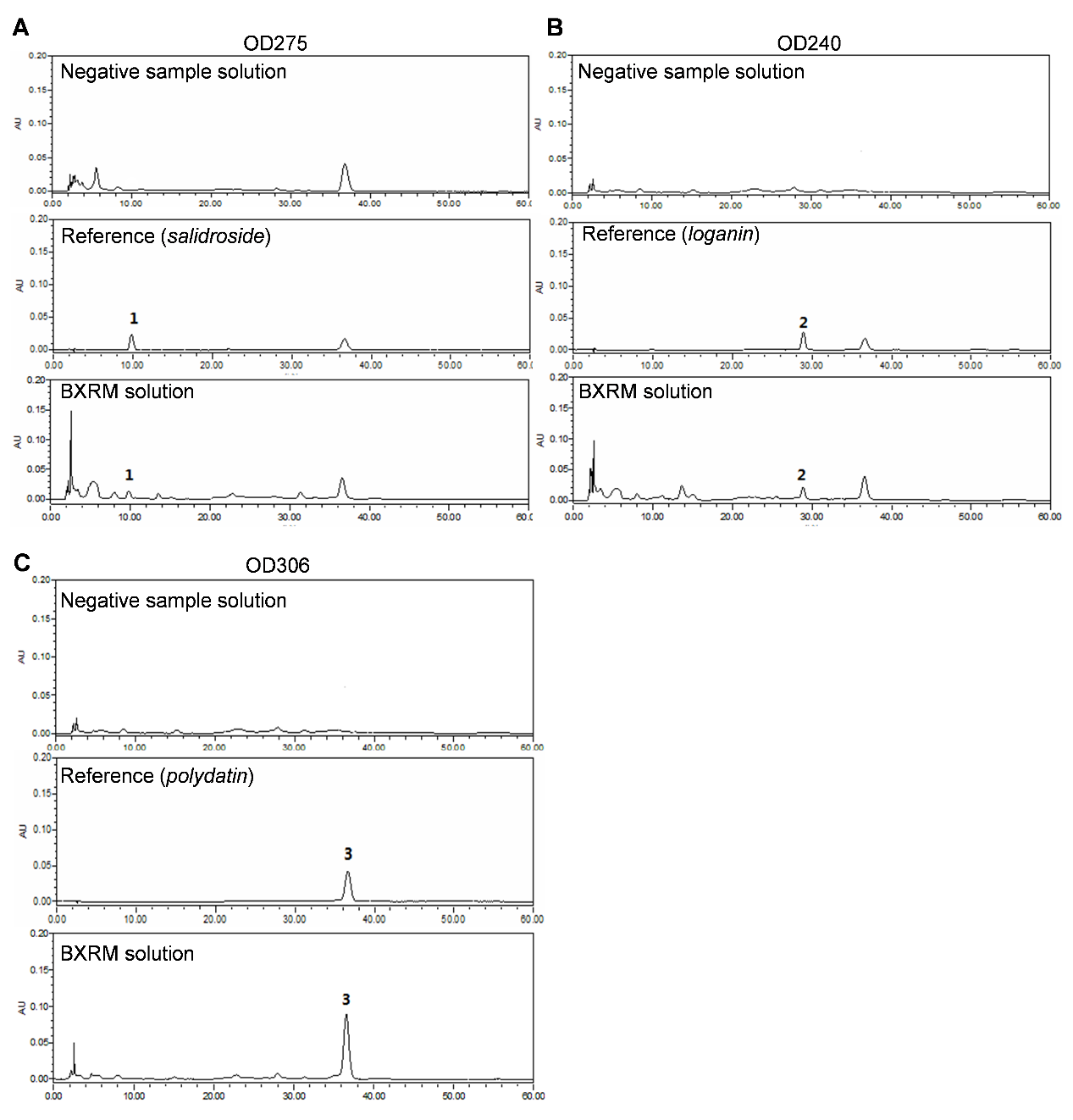

Supplement: Supplementary Materials — Supporting Figure 1: the compositions of Granule of BU-XIN RUAN-MAI. In this study, the compositions of Granule of BU-XIN RUAN-MAI were determined by LC-QTOF-MS analysis. The results showed that salidroside (a), loganin (b), and polydatin (c) were the main compounds of Granule of BU-XIN RUAN-MAI granule. [file 1808419.f1.docx]
